# Artificial Intelligence for Thyroid Nodule Characterization: Where Are We Standing?

**DOI:** 10.3390/cancers14143357

**Published:** 2022-07-10

**Authors:** Salvatore Sorrenti, Vincenzo Dolcetti, Maija Radzina, Maria Irene Bellini, Fabrizio Frezza, Khushboo Munir, Giorgio Grani, Cosimo Durante, Vito D’Andrea, Emanuele David, Pietro Giorgio Calò, Eleonora Lori, Vito Cantisani

**Affiliations:** 1 Department of Surgical Sciences, “Sapienza” University of Rome, 00161 Rome, Italy; salvatore.sorrenti@uniroma1.it (S.S.); vito.dandrea@uniroma1.it (V.D.); eleonora.lori@uniroma1.it (E.L.); 2 Department of Radiological, Anatomo-Pathological Sciences, “Sapienza” University of Rome, 00161 Rome, Italy; vincenzodolcetti@gmail.com (V.D.); vito.cantisani@uniroma1.it (V.C.); 3 Radiology Research Laboratory, Riga Stradins University, LV-1007 Riga, Latvia; mradzina@gmail.com; 4 Medical Faculty, University of Latvia, Diagnostic Radiology Institute, Paula Stradina Clinical University Hospital, LV-1007 Riga, Latvia; 5 Department of Information Engineering, Electronics and Telecommunications, “Sapienza” University of Rome, 00184 Rome, Italy; fabrizio.frezza@uniroma1.it (F.F.); khushboo.muniruniroma1@gmail.com (K.M.); 6 Consorzio Nazionale Interuniversitario per le Telecomunicazioni (CNIT), Viale G.P. Usberti 181/A Sede Scientifica di Ingegneria-Palazzina 3, 43124 Parma, Italy; 7 Department of Translational and Precision Medicine, “Sapienza” University of Rome, 00161 Rome, Italy; giorgio.grani@uniroma1.it (G.G.); cosimo.durante@uniroma1.it (C.D.); emanuele.david@uniroma1.it (E.D.); 8 Department of Surgical Sciences, “Policlinico Universitario Duilio Casula”, University of Cagliari, 09042 Monserrato, Italy; pgcalo@unica.it

**Keywords:** artificial intelligence, machine learning, thyroid cancer

## Abstract

**Simple Summary:**

In the present review, an up-to-date summary of the state of the art of artificial intelligence (AI) implementation for thyroid nodule characterization and cancer is provided. The opinion on the real effectiveness of AI systems remains controversial. Taking into consideration the largest and most scientifically valid studies, it is possible to state that AI provides results that are comparable or inferior to expert ultrasound specialists and radiologists. Promising data approve AI as a support tool and simultaneously highlight the need for a radiologist supervisory framework for AI provided results. Therefore, current solutions might be more suitable for educational purposes.

**Abstract:**

Machine learning (ML) is an interdisciplinary sector in the subset of artificial intelligence (AI) that creates systems to set up logical connections using algorithms, and thus offers predictions for complex data analysis. In the present review, an up-to-date summary of the current state of the art regarding ML and AI implementation for thyroid nodule ultrasound characterization and cancer is provided, highlighting controversies over AI application as well as possible benefits of ML, such as, for example, training purposes. There is evidence that AI increases diagnostic accuracy and significantly limits inter-observer variability by using standardized mathematical algorithms. It could also be of aid in practice settings with limited sub-specialty expertise, offering a second opinion by means of radiomics and computer-assisted diagnosis. The introduction of AI represents a revolutionary event in thyroid nodule evaluation, but key issues for further implementation include integration with radiologist expertise, impact on workflow and efficiency, and performance monitoring.

## 1. Introduction

For thyroid nodule management, the current diagnostic goal is early identification of the malignant thyroid nodules: although the incidence of the disease is high (incidence rate of 3.4/100,000 in men and 11.5/100,000 in women [1]), more than half of newly diagnosed thyroid cancers have a low risk of persistence or recurrence [2,3]. It is therefore necessary to develop a diagnostic tool that improves interobserver agreement in the risk stratification of thyroid nodules to provide an objective assessment of utility for the clinical and surgical management phases that follow [4], given that even molecular biology is not specific and does not accurately predict prognosis after surgery [5,6].

In the last two decades, medical imaging has grown exponentially, shifting from the traditional use of images for visual interpretation to their conversion to quantitative features that can be analyzed to extrapolate data and thus improve clinical decision-making. This approach is usually called “Radiomics” [7,8]. Radiomics takes advantage from extraction algorithms to derive several quantitative features from radiological images. Several recent works underline how these data may be used by machine learning (ML) systems.

**Figure 1:**
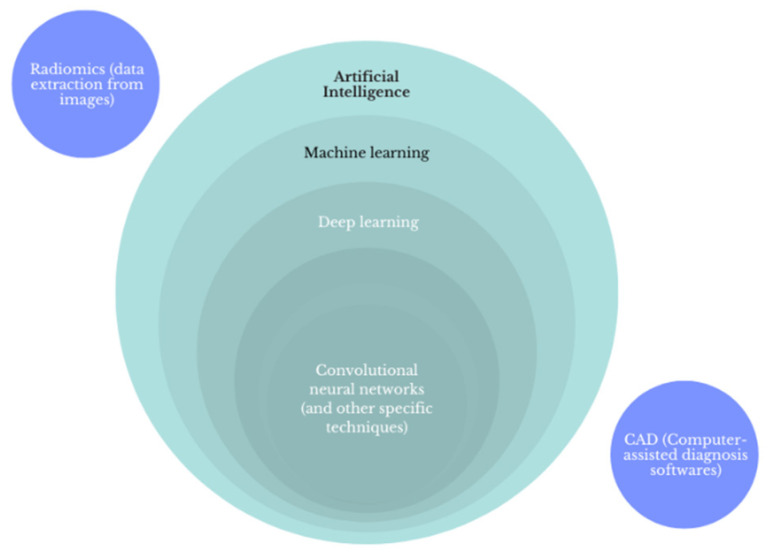
Schematic definition of artificial intelligence, machine learning, deep learning, and convolutional neural networks.

ML is an interdisciplinary sector in the subset of artificial intelligence (AI) dealing with the creation of systems that set up logical connections via algorithms to make predictions on data systems [9], Figure 1. The most interesting application of ML in the medical field is the discernment of patterns based on the examination and analysis of extensive datasets coming from various sources (clinical databases, laboratory results, and imaging data) [10,11]. In particular, ML techniques are divided into supervised and unsupervised learning methods. Supervised ML uses dataset inputs linked to dataset (labeled) outputs to identify a function between the two, while unsupervised ML uses non-labeled input datasets to identify and separate subsets with similar characteristics [12].

Deep learning (DL) is subset of ML approaches that uses neural networks arranged in layers to extract higher level features from input data and automatically learn their discriminative features, which allows approximation of non-linear relationships with excellent performance.

These technologies may be finally transferred to software used directly by clinicians: Computer Aided Diagnosis (CAD). Such software can be stand-alone or integrated in sonographic equipment and help in the detection and evaluation of thyroid nodules, one of the most common endocrine diseases, with incidental finding on ultrasound (US) examination, especially in patients over 65 years of age [13].

## 2. Materials and Methods

The study only considered articles published in the last decade (2012–2022), since most of the literature concerning AI application in radiology has undergone extensive development only recently. Among these, only large retrospective and prospective studies, systematic reviews, and meta-analyses were selected, as overall, they have greater statistical significance. The research was carried out by interrogating the PubMed and Google Scholar online databases using the Mesh terms “thyroid nodule and artificial intelligence”, with the MESH terms present in the titles or abstracts. Only human studies were selected. The search identified 166 studies from January 2012 to April 2022; of these, 63 were further considered. After a full text read, the final studies included in the review were 30 in number; they are all listed below in Table 1 [14,15,16,17,18,19,20,21,22,23,24,25,26,27,28,29,30,31,32,33,34,35,36,37,38,39,40,41,42].

## 3. Results

### 3.1. Radiomics

Medical radiomics employs high-throughput automated extraction algorithms to obtain a large number of quantitative characteristics from image datasets and is able to identify measurable information that clinical evaluation alone cannot detect [12,43].

Two of the first radiomics approaches in thyroid nodule characterization were texture analysis and US echo-intensity evaluation [44]. The latter is affected by several factors, such as gain, dynamics, operator dependency, and probe variability, as well as by the US equipment performance. The diagnostic value of echo-intensity obtained by direct measurement is limited; however, the echo intensity of the nodule and surrounding tissues increases or decreases simultaneously when these factors alternate [45]. Therefore, the echo intensity of the thyroid nodule can be indirectly quantified by measuring the grayscale ratio of the nodule to the surrounding thyroid tissues, which is more objective than the subjective assessment [44,45,46]. In a pivotal single-center study, it was demonstrated that the ratio was significantly lower in malignant nodules compared to benign ones [46], while the ratio of the nodule to the strap muscle was influenced by gender and less clinically discriminant. The inter-rater agreement was fair (k = 0.40) for hypo-echogenicity, whereas it was substantial for the ratio (k = 0.74), confirming the reduction in variability. This approach was subsequently replicated by other groups, showing that, as suggested, the ratio may distinguish anechoic and markedly hypoechoic nodules [47], and if it is applied to different nodule sizes [48], software can differentiate between benign and malignant nodules [49], even in different settings [45]. One of the most significant examples is the multicenter study conducted by Liang et al., in which a radiomic score was compared with a score based on the ACR TI-RADS criteria (which take into account, in addition to the difference in echogenicity, characteristics such as composition, shape, margin, and echogenic foci), showing a close correlation between the latter and the assessment carried out by the AI [50]. Radiomics approaches using grayscale histogram and other more complex image analyses were furthermore proved to predict BRAF mutational status [51], lateral lymph node metastasis [52], and a disease-free survival term.

### 3.2. Deep Learning and Machine Learning and TIRADS Systems

Deep learning (DL) is one ML method that relies on networks of computational units (i.e., neural units arranged in layers that gradually extract higher-level features from input data and automatically learn discriminative features from data) that allow approximation of complex non-linear relationships with outstanding performance. DL can achieve diagnosis automation, avoiding human intervention. In medical applications, DL algorithms are implemented for detection and characterization of tissue lesions as well as for the analysis of disease progression [12].

AI has already been widely used in thyroid imaging [11,53]. Several AI and ML approaches were implemented for the classification of thyroid nodules and the early detection of cancers, including modifications to the American College of Radiology Thyroid Imaging Reporting and Data System (TIRADS) systems that may be manually applied. Furthermore, a convolutional neural-network-based CAD program may help in predicting the BRAFV600E genetic mutation [54,55,56].

**Figure 2:**
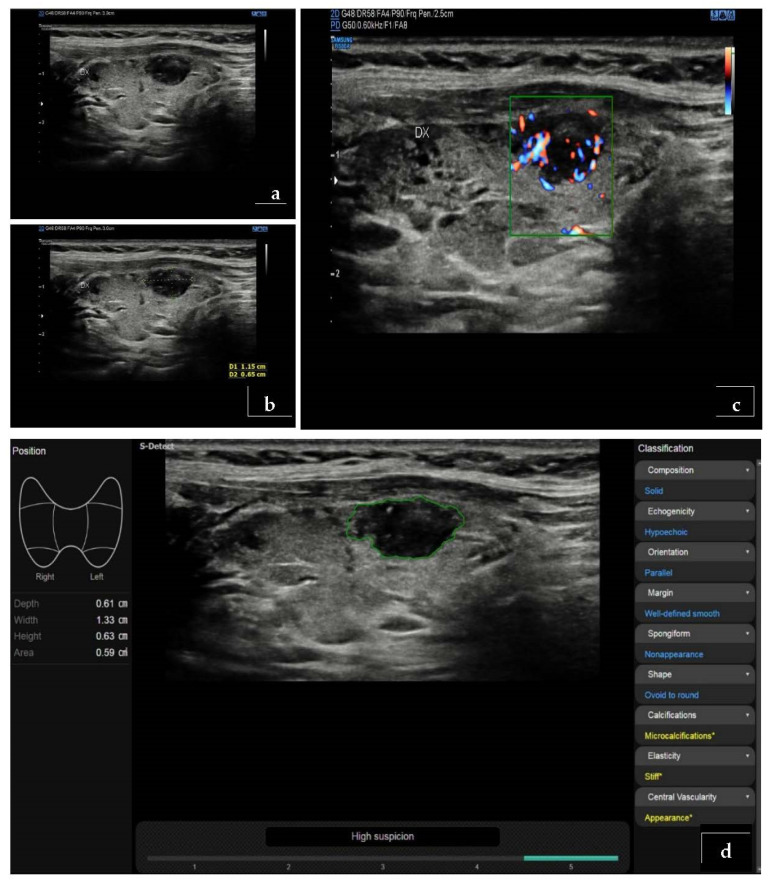
Histologically proved papillary carcinoma. (**a**,**b**) At B-mode, the nodule appears markedly hypoechoic. (**c**) At ColorDoppler-US evaluation, it shows type III vascular pattern (intra- and perinodular). (**d**) The evaluation with S-detect software (Samsung Medison, Co., Ltd., Seoul, Korea) confirms the high degree of suspicion (K-TIRADS 5).

**Figure 3:**
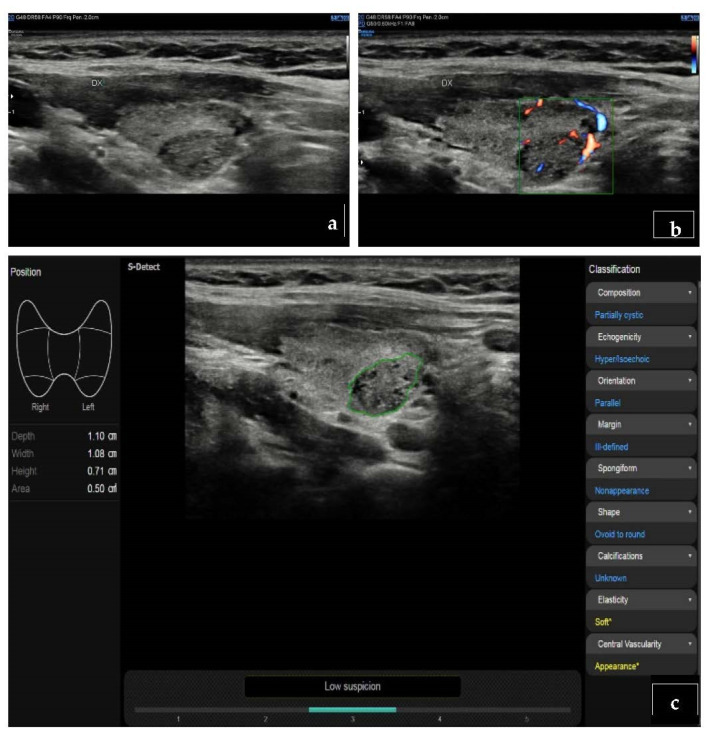
Histologically proved follicular hyperplasia. (**a**) The nodule appears iso-hypoechoic at B-mode. (**b**) At ColorDoppler-US evaluation, it shows type III vascular pattern (intra- and perinodular) (**c**) At S-detect software, the nodule is classified as low degree of suspicion (K-TIRADS 3).

Use of the ML approach may also identify nodules with high-risk mutations on molecular testing [57]. Another important advantage of AI systems is the possibility to obtain more systematized results, which could reduce inter-observer variability and tend to standardize the results obtained through the application of different TIRADS classification systems, whose major limit to date is represented by highly variable predictive capacity, high heterogeneity in grading, and the absence of reliable data in small nodules (<10 mm) [3,58,59] (Figure 2 and Figure 3). A recent TIRADS model showed higher accuracy than a model based on training according to the nodule status, i.e., benign and malignant; additionally, the specificity of the above-mentioned model was higher than that of both experienced and junior radiologists [60]. Comparisons between different imaging modalities are represented in Figure 2 and Figure 3, where a DL-based software confirms the suspect based on B-mode US imaging.

### 3.3. Computer-Assisted Diagnosis (CAD)

These approaches may produce new knowledge by identifying new patterns and features to be applied in a more traditional way and generating computer-assisted diagnosis (CAD) systems; i.e., software able to analyze data through the application of machine-learning principles to aid clinicians for a “second opinion” provision. AI-based thyroid CADs may further improve diagnostic performance and reliability, reaching an accuracy similar to that obtained by an expert radiologist [10,11], with potential implication in training of less-experienced operators and reduction of intra- and inter-observer variability [11].

CAD-systems are already available as commercial applications or where embedded in US equipment. A recent meta-analysis [61] confirmed that their performance in evaluating malignant thyroid nodules is comparable to radiologists. Specifically, the sensitivity was reported to be like that of experienced radiologists, while specificity and diagnostic odds ratio were reduced [39]. While these systems did not outperform experienced specialists, they are able to guide the training of less-skilled examiners, thus reducing variability when clinician’s judgements show significant disagreement. However, it is difficult to eliminate all possible sources of inter-observer variability: it is in fact possible that radiologists with different degrees of experience select images with more or less relevant characteristics of suspicion. The homogeneity of the image segmentation process also plays a fundamental role in reducing the impact of selection bias. The segmentation process in fact involves a manual selection of the area of interest (which should correspond to the nodule), but in this phase it is possible that portions of the slide that contain non-informative areas are selected, compromising the training process of the AI system. To try to solve the problem, some studies have adopted a two-step fully automated classification system, specifically trained both to autonomously select the area of interest and to predict the final pathology of the specific selected area [62]

Furthermore, the models generated by images obtained from different machines may not be universally generalizable, which can determine limits in the sampling phases and in the standardization of software. This therefore requires an accurate evaluation and selection phase prior to the adoption of an AI system in any case [11]. Table 2 summarizes main advantages and disadvantages of artificial intelligence over conventional imaging. 

## 4. Discussion

The TIRADS system was developed to improve the diagnostic accuracy of conventional US in thyroid nodule characterization [63]. However, its clinical use is still very limited and diverse; in particular, there are various types of TIRADS, and their application is very subjective; therefore, it is significantly affected by inter-observer variability [64].

AI could increase US accuracy and significantly limit inter-observer variability by using standardized mathematical algorithms. In the world of DL, many authors are focusing on convolutional neural networks (CNNs), introduced by LeCun [65,66]. Before their diagnostic accuracy can be assessed, CNNs are trained by subjecting them to specific algorithm-segmented US images of thyroid nodules with known histological diagnosis; at the end of the learning phase the CNNs are able to analyze the captures of thyroid nodules and to suggest a risk stratification of these nodules in correlation to a specific TI-RADS level [16]. Most of the existing literature evaluates the diagnostic accuracy of various types of properly trained convolutional neural networks by comparing them to those of radiologists with variable degrees of experience. All the evaluated studies showed significant high overall diagnostic accuracy of CNNs, above 90%, which does not differ much from that of expert radiologists. In particular, most of the studies demonstrate a comparable diagnostic accuracy, such as Watkins et al., Bai et al., Ye et al., Koh et al., and Fresilli et al. [4,16,20,30,40]. Approximately the same number of studies demonstrate a higher diagnostic accuracy of AI systems compared to that of expert radiologists (e.g., Sun et al., Peng et al., and Zhou et al.) [15,22,23], or vice versa, a superiority of diagnostic accuracy by expert radiologists compared to that of AI systems (e.g., Zhang et al. and Han et al.) [32,33]. Despite controversial results, the meta-analysis conducted by Zhao et al. suggests that the sensitivity of the CAD system is like that of experienced radiologists, but the CAD system has lower specificity and diagnostic odds ratio than experienced radiologists [39].

On the other hand, almost all the studies included in this review show that CNNs obtain a better result than junior radiologists with less than 5 years of experience in US evaluation of thyroid nodules [4,23,34,40], especially with regards to specificity [60]. These studies therefore agree in suggesting that CAD systems may be an effective support tool to increase the diagnostic efficacy of thyroid nodule evaluations by less-experienced radiologists [25]. Furthermore, some studies, such as the one by Zhao et al., show that the diagnostic accuracy of senior radiologists assisted by CAD systems is higher than that of radiologists alone and CAD systems alone [39].

It is therefore not yet clear from the literature analysis which of the specific AI systems has the best diagnostic accuracy. Wang et al. compare the effectiveness of only few CNNs [25], while most studies analyze specific systems individually, showing high specificity—especially if they are based on TIRADS system algorithm—rather than differentiation among benign and malignant nodules with surgical histopathological reference [60]. In absolute terms, the CAD system used by Zhou et al., a CNN-based transfer learning method named DLRT (deep-learning radiomics of thyroid), appears to be one of those with greater diagnostic accuracy (AUC 0.97) [23], although this type of comparison between AI systems has no real statistical significance as they were analyzed on retrospective datasets.

In addition, a variety of AI technologies have been evaluated on thyroid cytology specimens. Unfortunately, no application has been demonstrated to be robust enough for clinical use in FNAB result analysis, an issue which is related to the multi-layered, multi-dimensional, complex interpretation process and the lack of standardized algorithms [66,67]. However, Ippolito et al. [68] show collaborative data between cytology and US; they integrated microscopic pathology characteristics, clinical data, and imaging features into a combined algorithm to triage indeterminate and follicular lesions into high- or low-risk categories using a CNN framework that demonstrated a sensitivity of 85.7% and low specificity of 58.8%. As an element of evidence that emerged from the present review, key issues in AI implementation include integration with radiologist interpretation, impact on workflow and efficiency, and performance monitoring. This can be translated into an automated structured report for integration into a radiology report. Sensitivity settings for different features can be adjusted and customized; validation by an experienced radiologist co-reader is warranted [69].

AI tools may be useful in practice settings with limited subspeciality expertise: using AI solutions in the settings with minimal radiology support and high negative predictive value may provide comfort for clinicians with no need for follow-up of benign findings, although this should be addressed with caution. Depending on the institutional cohorts, AI results cannot be generalized, as it is assumed that AI would misperform in specialized centers with higher malignancy rates in comparison to the average population [69]. In terms of legal frame, AI-generated conclusions being reviewed by board-certified radiologists or US practitioners, regardless of their specialty, is mandatory. Several authors suggest use of AI results as second-opinion, although this has a negative impact on workflow speed [10,11,69]. US practices, in conjunction with vendors, should implement AI performance and quality control protocols in order to assess the reliability of the tool.

Finally, a limitation of AI should be noted: thyroid US scanning includes comprehensive neck soft tissue assessment, including lymph nodes and parathyroid glands, but currently, AI solutions address only one aspect of this complex examination.

## 5. Conclusions

The introduction of AI was a revolutionary event in thyroid nodule assessment. Not only ultrasound, but also other imaging methods such as CT and MRI, use it effectively [70,71,72]. In some cases, there is even the possibility to effectively predict the immunohistochemistry of the thyroid nodule simply through the evaluation of segmented image datasets by AI systems [73]. Moreover, the use of CAD in daily clinical practice does not have a significant impact on workflow, as it increases the examination time by approximately 2–3 min [4]

However, the real effectiveness of AI systems remains controversial; taking into consideration the largest and most scientifically valid studies, it is possible to state that AI provides results that are comparable or in any case inferior to that of expert radiologists. Furthermore, it is necessary to consider the relevant heterogeneity of sensitivity and specificity between studies, due to the diversity in methodology and to the differences among patients included [39].

AI systems still have a long way to go to replace experienced radiologists in the process of improving accuracy and reducing time consumption, and larger studies meeting uniformity criteria are necessary to evaluate the diagnostic performance of these systems further. Nevertheless, the current CAD systems offer support for radiologists in thyroid nodule assessment and increase the overall accuracy in routine thyroid US [10,11,39].

AI solutions with CAD should be implemented in the teaching process of junior specialists. Deep-learning algorithms would benefit from follow-up US imaging data of the same thyroid nodules in combination with TIRADS classification, rather than dichotomous prediction, to increase their repeatability, reliability, and accuracy.

Regarding the legal frame, AI-generated conclusions should be reviewed by board-certified radiologists or US practitioners as mandatory practice, such that AI results may be provided only as a second opinion.

## Figures and Tables

**Table 1:** Machine learning approaches for the evaluation of thyroid nodule sonographic images.

Reference	Approach	Source Data	Method Details	Performance
Zhu, et al., 2021 [14]	Brief Efficient Thyroid Network (BETNET; a CSS model)	gray-scale US images of 592 patients with 600 TNs (internal dataset) 187 patients with 200 TNs (external validation dataset)	CNN approach with 24 layers: 13 convolution layers, 5 pooling layers, 3 fully connected layers with dropouts in between	AUC 0.970, 95% CI: 0.958–0.980 in the independent validation cohort; similar to two highly skilled radiologists (0.940 and 0.953)
Peng, et al. 2021 [15]	Deep-learning AI model (ThyNet)	18,049 US images of 8339 patients (training set) 4305 images of 2775 patients (total test set)	combined architecture of three networks: ResNet, ResNeXt, and DenseNet	ThyNet AUC (0.922; 95% CI 0.910–0.934] higher than that of the radiologists (0.839; CI 0.834–0.844]; *p* < 0.0001)
Bai, et al., 2021 [16]	RS-Net evaluation AI model	13,984 thyroid US images	CNN approach in which GoogLeNet is used as the backbone network.	Accuracy, sensitivity, specificity, PPV, and NPV were 88.0%, 98.1%, 79.1%, 80.5%, and 97.9%, comparable to that of a senior radiologist
Yoon, et al., 2021 [17]	Texture analysis; least absolute shrinkage and selection operator (LASSO) logistic regression model including clinical variables	155 US images of indeterminate thyroid nodules in 154 patients.	Texture extraction using MATLAB 2019b.; the LASSO model was used to choose the most useful predictive features. Univariable and multivariable logistic regression analyses were performed to build malignancy prediction models.	Integrated model AUC 0.839 vs. 0.583 (clinical variables only).
Liu, et al., 2021 [18]	information fusion-based joint convolutional neural network (IF-JCNN)	163 pairs of US images and raw radiofrequency signals of thyroid nodules	IF-JCNN contains two branched CNNs for deep feature extraction: one for US images (14 convolutional layers and 3 fully connected layers) and the other one for RF signals (12 convolutional layers and 3 fully connected layers)	The information carried by raw radiofrequency signals and ultrasound images for thyroid nodules is complementary IF-JCNN (both images and RF signals): AUC 0.956 (95% CI 0.926–0.987)
Gomes Ataide, et al., 2020 [19]	Feature extraction and Random Forest classifier	99 original US images	Feature extraction using MATLAB 2018b; Random Forest classifier (400 Decision Trees; Criterion: Entropy, with Bootstrap)	RFC accuracy 99.3%, sensitivity 99.4%, specificity 99.2%
Ye, et al., 2020 [20]	Deep convolution neural network (VGG-16)	US images of 1601 nodules (training set) and test data including 209 nodules (test set)	CNN approach based on VGG-19 (16 layers with learnable weights, 13 convolutions and 3 fully connected layers)	AUC 0.9157, comparable to the experienced radiologist (0.8879; *p* > 0.1)
Wei, et al., 2020 [21]	Ensemble deep learning model (EDLC-TN)	25,509 thyroid US images	CNN model based on DenseNet and adopted as a multistep cascade pathway for an ensemble learning model with voting system.	AUC 0.941 (0.936–0.946)
Zhou, et al., 2020 [23]	CNN-based transfer learning method named DLRT (deep-learning radiomics of thyroid)	US images of 1750 thyroid nodules (from 1734 patients)	CNN-based architecture with transfer learning strategy, with 4 hidden layers (3 transferred and a fine-tuned layer) and a fully connected layer	AUC in the external cohort 0.97 (0.95–0.99). Both a senior and a junior US radiologist had lower sensitivity and specificity than DLRT.
Nguyen, et al., 2020 [24]	Combination of multiple CNN models (ResNet-based and InceptionNet-based)	450 US thyroid nodule images (from 298 patients)	Combination of ResNet50-based (50 layers) and Inception-based (4 layers) networks followed by global average pooling, batch normalization, dropout, and dense layer	Accuracy: 92.05%
Wang, et al., 2020 [25]	Three CNN networks (feature extraction network; attention-based feature aggregation network; classification network)	7803 US thyroid nodule images from 1046 examinations	CNN approach based on Inception-Resnet-v2 (164 layers)	Method AUC 0.9006 Both the accuracy and sensitivity are significantly higher than sonographers.
Thomas, et al., 2020 [26]	AIBx, AI model to risk stratify thyroid nodules	2025 US images of 482 thyroid nodules (internal dataset) and 103 nodules (external dataset)	CNN approach based on ResNet 34 (34 layers)	Negative predictive value (NPV), sensitivity, specificity, positive predictive value (PPV), and accuracy of the image similarity model were greater than other cancer risk stratification systems.
Galimzianova, et al., 2020 [27]	Feature extraction and regularized logistic regression model	92 US images of 92 biopsy-confirmed thyroid nodules	Feature extraction (219 for each nodule) and elastic net regression analysis	Method AUC 0.828 (95% CI, 0.715–0.942), greater than or comparable to that of the expert classifiers
Nguyen, et al., 2019 [28]	AI-Based Thyroid Nodule Classification Using Information from Spatial and Frequency Domains	ultrasound thyroid images of 237 patients (training dataset) and 61 patients (test dataset).	CNN models (Resnet18, Resnet34, and Resnet50 were compared)	AI system with spatial domain based on deep learning, and frequency domain based on Fast Fourier transform (FFT) outperforms the state-of-the-art methods (especially CAD systems)
Buda, et al., 2019 [29]	CNN	1377 US images of thyroid nodules in 1230 patients (training dataset) and 99 nodules (internal test dataset)	Custom CNN (six blocks with 3 × 3 convolutional filters, followed by Rectified Linear Unit activation function and max pooling layer with 2 × 2 kernels).	Method AUC: 0.87 [CI 0.76, 0.95] Three ACR-TIRADS readers 0.91
Koh, et al., 2020 [30]	Two individual CNNs compared with experienced radiologist	15,375 US images of thyroid nodules (training set), 634 (internal test), 1181 (external test set).	Four CNNs including two individual CNNs, ResNet50 (50 layers) and InceptionResNetV2 (164 layers), and two classification ensembles, AlexNet-GoogLeNet-SqueezeNet ensemble and AlexNet-GoogLeNetSqueezeNet-InceptionResNetv2 ensemble	CNNs AUC similar to experienced radiologist AUC (0.87)
Wang, et al., 2019 [31]	CNN compared with experienced radiologist	351 US images with nodules and 213 images without nodules of 276 patients	CNN system in which the Resnet v2-50 (50 layers) network and YOLOv2 are integrated	CAD AUC 0.902 significantly higher than radiologist AUC 0.859 (*p* = 0.0434)
**CAD systems**				
Sun, et al., 2020 [22]	Fused features combing the CNN-based features (VGG F-based features) with hand-crafted features	1037 US images of thyroid nodules (internal dataset) and 550 images (test dataset)	A support vector machine (SVM) is used for classification and fused features which combined the deep features extracted by a CNN with hand-crafted features, such as the histogram of oriented gradient (HOG), local binary patterns (LBP), and scale invariant feature transform (SIFT)	AUC of attending radiology lower than system (0.819 vs. 0.881, *p* = 0.0003)
Han, et al., 2021 [32]	S-Detect for Thyroid	US images of 454 thyroid nodules from 372 consecutive patients	S-Detect for Thyroid is an AI-based CAD software integrated in US equipment (Samsung Medison Co., Seoul, South Korea)	The sensitivities of the CAD system did not differ significantly from those of the radiologist (all *p* > 0.05); the specificities and accuracies were significantly lower than those of the radiologist (all *p* < 0.001).
Zhang, et al., 2020 [33]	AI-SONIC; Demetics Medical Technology Co., Zhejiang, China	US images of 365 thyroid nodules	AI-SONIC is a CAD based on deep learning (cascade CNN of two different CNN architectures (one with 15 convolutional layers/2 pooling layers for segmentation, and the other with 4 convolutional layers/4 pooling layers for detection), developed by Demetics Medical Technology Co., China	AUC CAD 0.788 vs. senior radiologist 0.906, *p* < 0.001). The use of CAD system improved the diagnostic sensitivities of both the senior and the junior radiologists
Fresilli, et al., 2020 [4]	S-Detect for Thyroid compared with an expert radiologist, a senior resident and a medical student evaluation	US images of 107 thyroid nodules	S-Detect for Thyroid is an AI-based CAD software integrated in US equipment (Samsung Medison Co., Seoul, South Korea)	The CAD system and the expert achieved similar values of a sensitivity and specificity (about 70%–87.5%). The specificity achieved by the student was significantly lower (76.25%).
Jin, et al., 2020 [34]	CAD system based on a modified, CNN-based TIRADS, evaluated by	US images of 789 thyroid nodules from 695 patients	CAD system based on the ACR TI-RADS automatic scoring using a CNN (no details provided).	AUC CAD 0.87 AUC Junior radiologist 0.73 (Junion + CAD): 0.83 AUC Senior radiologist 0.91
Xia, et al., 2019 [35]	S-Detect for Thyroid	US images of 180 thyroid nodules in 171 consecutive patients	S-Detect for Thyroid is an AI-based CAD software integrated in US equipment (Samsung Medison Co., Seoul, South Korea)	AUC CADs 0.659 (0.577–0.740) AUC radiologist 0.823 (0.758–0.887)
Jin, et al., 2019 [36]	AmCad; AmCad BioMed, Taipei City, Taiwan	33 images from 33 patients read by 81 radiologists	Commercial standalone CAD software: AmCad (version: Shanghai Sixth People’s Hospital; AmCad BioMed, Taipei City, Taiwan)	CAD AUC 0.985 (0.881–1.00) 177 contestants AUC 0.659 (0.645–0.673) (*p* < 0.01)
Kim, et al., 2019 [37]	S-Detect for Thyroid 1 and 2	US images of 218 thyroid nodules from 106 consecutive patients	S-Detect for Thyroid is an AI-based CAD software integrated in US equipment (Samsung Medison Co., Seoul, South Korea)	AUC: radiologist 0.905 (95% CI, 0.859–0.941) S-Detect 1–assisted radiologist 0.865 (0.812–0.907) S-Detect 1 0.814 (0.756–0.863) S-Detect 2-assisted radiologist 0.802 (0.743–0.853) S-Detect 2 0.748 (0.685–0.804)
Chi, et al., 2017 [38]	CAD system for thyroid nodule	Database 1 includes 428 images in total while database 2 includes 164 images in total	CAD based on fine tuning of GoogLeNet CNN (22 convolutional layers including 9 inception modules)	CAD AUC 0.9920 Experienced radiologist AUC 0.9135
Zhao, et al., 2019 [39]	CAD system for thyroid nodule systematic review and meta-analysis	Meta-analysis of 5 studies with 723 thyroid nodules from 536 patients	4 studies with S-Detect; 1 study with internal CAD based on CNN.	CAD AUC 0.90 (95% CI 0.87–0.92) Experienced radiologist AUC 0.96 (95% CI 0.94–0.97)
**AI-modified TIRADS**				
Watkins, et al., 2021 [40]	AI-TIRADS	US images of 218 nodules from 212 patients	The AI-TIRADS is an optimization of ACR TIRADS generated by “genetic algorithms”, a subgroup of AI methods that focus on algorithms inspired by “natural selection”.	Sensitivity 93.44% Specificity 45.71% BTA, ACR-TIRADS, and AI-TIRADS have comparable diagnostic performance
Wang, et al., 2020 [41]	Google AutoML for automated nodule identification and risk stratification	US images of 252 nodules from 249 patients.	Google AutoML algorithm (AutoML Vision; Google LLC), with cloud computing and transfer learning	Accuracy of 68.7 ± 7.4% of AI-integrated TIRADS
Wildman-Tobriner, et al., 2010 [42]	AI-TIRADS	US images of 1425 biopsy-proven thyroid nodules from 1264 consecutive patients (training set); 100 nodules (test set)	The AI-TIRADS is an optimization of ACR TIRADS generated by “genetic algorithms”, a subgroup of AI methods that focus on algorithms inspired by “natural selection”.	ACR TI-RADS AUC 0.91 AI TI-RADS AUC 0.93 (with slight improvement of specificity and ease of use)

Abbreviations: ACR: American College of Radiology; AI: artificial intelligence; AIBx: AI model to risk stratify thyroid nodules; AUC: area under the curve; AutoML: Auto machine learning; BETNET: brief efficient thyroid network; CAD: computer-aided diagnosis; CI: confidence interval; CNN: convolution neural network; CSS: cascading style sheets; DLRT: deep-learning radiomics of thyroid; EDLC-TN: ensemble deep-learning classification model for thyroid nodules; FFT: Fast Fourier transform; IF-JCNN: information fusion-based joint convolutional neural network; LASSO: Least Absolute Shrinkage and Selection Operator; NPV: negative predictive value; PPV: positive predictive value; RF: radiofrequency; RFC: Random Forest classifier; RS-NET: regression–segmentation network; US: ultrasound; VGG: Visual Geometry Group.

**Table 2:** Advantages and disadvantages of artificial intelligence over conventional imaging.

Main Advantages of AI	Main Disadvantages of AI
It is based on models, for the interpretation of thyroid nodules, that are able to match the performance characteristics of radiologists and pathologists	Too little experience at the moment; prospective multicenter trials on a wide population will be needed to improve the utility of artificial intelligence for the interpretation of thyroid nodules
Usable software for thyroid nodule risk stratification are already commercially available	
